# Interrogating domain-domain interactions with parsimony based approaches

**DOI:** 10.1186/1471-2105-9-171

**Published:** 2008-03-26

**Authors:** Katia S Guimarães, Teresa M Przytycka

**Affiliations:** 1 National Center of Biotechnology, National Library of Medicine, National Institutes of Health, Bethesda, MD 20894, USA; 2 Center of Informatics, Federal University of Pernambuco, Recife, PE 50732, Brazil

## Abstract

**Background:**

The identification and characterization of interacting domain pairs is an important step towards understanding protein interactions. In the last few years, several methods to predict domain interactions have been proposed. Understanding the power and the limitations of these methods is key to the development of improved approaches and better understanding of the nature of these interactions.

**Results:**

Building on the previously published Parsimonious Explanation method (PE) to predict domain-domain interactions, we introduced a new Generalized Parsimonious Explanation (GPE) method, which (i) adjusts the granularity of the domain definition to the granularity of the input data set and (ii) permits domain interactions to have different costs. This allowed for preferential selection of the so-called "co-occurring domains" as possible mediators of interactions between proteins. The performance of both variants of the parsimony method are competitive to the performance of the top algorithms for this problem even though parsimony methods use less information than some of the other methods. We also examined possible enrichment of co-occurring domains and homo-domains among domain interactions mediating the interaction of proteins in the network. The corresponding study was performed by surveying domain interactions predicted by the GPE method as well as by using a combinatorial counting approach independent of any prediction method. Our findings indicate that, while there is a considerable propensity towards these special domain pairs among predicted domain interactions, this overrepresentation is significantly lower than in the iPfam dataset.

**Conclusion:**

The Generalized Parsimonious Explanation approach provides a new means to predict and study domain-domain interactions. We showed that, under the assumption that all protein interactions in the network are mediated by domain interactions, there exists a significant deviation of the properties of domain interactions mediating interactions in the network from that of iPfam data.

## Background

### Introduction

Understanding of protein and domain interactions is necessary to comprehend the functioning of a cell. In the past few years, this area has been the subject of intensive study (surveyed in [1]) As the power and the limitations of methods to predict domain interactions become clear, new and improved approaches have been developed [2,3].

Protein interaction data is collected from studies of individual systems, and more recently through high-throughput experiments, such as yeast two-hybrid (Y2H) and tandem affinity purification followed by mass spectrometry (TAP/MS) [4-11]. Those methods provide a vast amount of interaction data, but several independent studies indicate false positive rates of the order of 50% [12-15]. This necessitates proper modeling of the presence of noise by the computational methods that use this data.

It has been estimated that more than half of eukaryotic proteins and a significant fraction (between one third and two thirds, depending on the estimates) of prokaryotic proteins are multi-domain proteins [16-19]. It is often assumed that the interaction between two proteins involves binding between two or more specific domains. Under this assumption, many research groups have contributed computational methods aimed at discovering interacting domains. The first such prediction method, the *Association *method [20], scores each domain pair by the ratio of the number of occurrences of a given pair in interacting proteins to the number of independent occurrences of those domains. Deng and colleagues [21] proposed an expectation maximization algorithm (*EM*) which computes domain interaction probabilities that maximize the expectation of observing a given protein-protein interaction network. Other approaches to this problem use linear programming [22], support vector machines [23], probabilistic network modeling [24], and lowest p-value [25].

More recently, Riley and colleagues [26] introduced a method, called the Domain Pair Exclusion Analysis (DPEA), which predicts domain interactions by computing, for every potentially interacting domain pair, the so called E-value. A modification of this method has been proposed lately in [27]. The E-value measures to what extent disallowing an interaction between the domains in a given pair reduces the likelihood of observing the protein interaction network. Domain pairs with E-value above a certain threshold are predicted as interacting. The idea that domain-domain interactions should be discovered as putative explanations of protein-protein interactions rather than predictors of these interactions was also the cornerstone of a recently proposed *Parsimonious Explanation *(*PE*) method [2], which assumes that interactions between proteins evolved in a parsimonious way, and uses optimization to predict domain interactions. Lee and colleagues [3] improved on a previous work [21] by creating what we refer to in this paper as the *Integrated Bayesian *(*IB*) method. This method estimates the likelihood of domain interactions based on a protein interaction network from four different organisms (prediction from each network is treated as independent evidence), and on the amount of biological evidence relating two domains, such as co-occurrence of domains in the same protein and existence of common GO terms at the functional level.

### Adjusting the granularity of domain definition

While there is no agreement on the definition of a domain, it is often assumed that domains are independent evolutionary units, in the sense that a domain is either observed in isolation in nature, or in the context of different multidomain protein architectures [28]. This definition inherently depends on the observed protein universe: as the number of proteins in the universe increases, we may obtain a finer partition into domains. Conversely, if we study a restricted set of proteins, it may be reasonable to use this set as the protein universe with respect to which we validate whether or not a given protein sequence is observed in isolation, or in more than one context in different multidomain proteins. In such a case, the granularity of the domain definition may be lower than in the full protein universe. In the context of predicting interactions between domains based on protein interaction data, it makes sense to adjust the granularity of the domain definition to the universe of proteins in the protein-protein interaction network. From a practical point of view, domains are typically assigned using Pfam HMM models [29] or a similar approach. Starting with Pfam assignment, we adjusted the granularity of the domain definition by unifying domains that are always seen together in our protein set into a so called *supra-domain*. The term supra-domain is borrowed from the work by Chothia and colleagues [30] where it was used to indicate a group of domains that appeared frequently together, albeit not necessarily always. A similar granularity adjustment was also made in [21].

### Parsimony principle

The parsimony principle (also known as Occam's razor) states that the explanation of any phenomenon should make as few assumptions as possible. In evolution, a parsimony approach seeks an explanation that requires the smallest number of evolutionary changes [31]. In the context of predicting domain interactions, the parsimony assumption is expressed as the hypothesis that the set of correct domain-domain interactions is well approximated by the minimal set of domain interactions necessary to justify a given protein interaction network. That idea was originally introduced in the PE method [2], and formulated as a linear programming optimization problem. In this formulation, each potentially interacting domain pair is represented by a variable, and each protein interaction defines a constraint enforcing that such interaction is "explained" (the fact that these interactions are not fully reliable is solved by a randomization procedure). The original PE method treats all domain interactions equally. However, just as in the general case of evolution, where some changes are more likely than others, some types of domain interactions may be preferred to others for biological reasons. To model this possibility we introduce a new variant of the parsimony approach, *Generalized Parsimonious Explanation *(*GPE*), which allows for a differential treatment of different types of domain pairs. It also adjusts the granularity of the domain definition by incorporating the supra-domains and the propensity towards predicting interactions between co-occurring domains.

### Co-occurring domains

It has been observed that domains which can be found in distinct protein chains in one organism whereas in a different organism they are fused together in one protein chain often interact [32,33]. This motivated Lee and colleagues to include co-occurrence (Lee and colleagues used the term "co-exist") of domain pairs in one protein chain as evidence of possible interaction between these domains [3]. Two domains are considered to be co-occurring if there is a protein chain that contains both domains. For example, if a protein contains domains A, B, and C then all three pairs (A, B), (A, C), and (C, B) are considered to be co-occurring. Using iPfam domain pairs as a gold standard, Lee and colleagues showed that the variant of the Expectation Maximization method based exclusively on information about domain co-occurrence gives more accurate predictions than the variant of this method based on interaction predicted independently in several organisms. This is interesting and we decided to investigate the reasons leading to this result in more detail. It is known that proteins in PDB are not representative of proteins encoded by the genomes. For example, they have different length distribution, amino-acid composition, distribution of predicted secondary structure type, level of disorder, etc. [17,34,35]. This prompted us to investigate whether statistical properties of domain pairs in iPfam are the same as those of interacting domain pairs mediating protein interactions in the high throughput genome scale interaction networks. Since the second set of interacting domain pairs is not available to us directly, we estimated properties of this set using two methods: by performing a survey of properties of predicted domain interactions, and by a combinatorial counting method independent of any prediction method.

### Benchmarking the prediction results

Due to limited availability of domain-domain interaction data, developing and benchmarking of domain interaction prediction methods is particularly challenging. A standard solution in the field is to use as a benchmark set interacting domain pairs obtained from crystal structures of protein complexes and collected in the iPfam database [36]. However, one needs to keep in mind that iPfam represents only a small fraction of interacting domain pairs. According to a recent study involving E. coli, yeast, worm, fly, and human data, conducted by Itzhaki and colleagues [37], the percentage of protein-protein interactions that can be explained by domain-domain interactions from iPfam or 3DID is no more than 20% for any of the organisms. Therefore, any domain interaction prediction method that undertakes the task of explaining protein interactions through domain-domain interactions is expected to (correctly) recover interacting domain pairs that are not in those high-confidence databases (yet). In a ROC-type analysis these interactions are typically incorrectly counted as false positives. Therefore, to evaluate the performance of various methods, we used a different method pioneered by Nye and colleagues [2,25,38]. This method considers only those interacting protein pairs that contain an iPfam domain pair as a possible explanation. Then, for every interacting protein pair, it tests if the corresponding iPfam pair is recovered as the highest scoring domain pair among all domain pairs that could potentially mediate this protein interaction. Using this approach, we compared the predictions of GPE with those of the PE method. We also compared the performances of GPE and the best-known methods for which the corresponding data was available.

## Results and Discussion

### Implementation of the Generalized Parsimonious Explanation Method

The parsimonious explanation model seeks the smallest set of domain interactions that can explain all protein interactions in the network. The original parsimonious explanation method [2] treated all possible domain pairs equally. In contrast, the Generalized Parsimonious Explanation (GPE) allows for incorporating a priori knowledge that some domain interactions may be preferable to others. This is done by modifying the objective function in the linear programming (LP) formulation of the associated optimization problem. We used this capacity of GPE to include bias towards interactions between domains that co-occur in the same protein chain and to test the impact of such bias on the predictions. Namely, the domain pairs that are found to co-occur in the same protein chains are assumed to have lower cost than other domain pairs.

The second difference between PE and GPE is the "granularity" of domain definition. Some domains have been fused together into one supra-domain according to the rules described above. For example, UreE urease accessory protein, C-terminal domain UreE_C is always observed together with UreE urease accessory protein, N-terminal domain UreE_N, and therefore these two domains are combined into one supra-domain. Additional file 1 contains a list of the created supra-domains and the associated list of original domains contained in each supra-domain. Supra-domains inherit interactions of the domains they contain. The benchmark set, modified from the original iPfam benchmark set using these rules, is available as Additional file 2. To distinguish between the original Pfam domains and our new set that contains also supra-domains, we use the term "generalized domains" when referring to our new set.

Similarly to the original PE method, GPE models the reliability of the edges in the protein interaction network using a randomized approach. This is done by constructing of a set of linear programming instances in a probabilistic fashion and averaging the results (for details see Methods). Two types of scores are reported: the LP-score and pw-score.

The LP score is a value between zero and one and is computed by averaging the outputs from the set of randomized linear programs. Note that if we additionally enforce that the solution to our linear program is integer then, for each domain pair, only two values would be possible: 0 – indicating that domain pair is not a part of an optimal solution and 1 – indicating otherwise. The real valued solution measures, for each domain pair, the contribution of a given domain pair to the optimal solution where high scores correspond to high contribution. The pw-score combines the traditional p-value (obtained via additional simulations) and the so called witness-score. The need for this additional witness score is a consequence of the following observation. Pairs of frequently occurring domains usually have high p-values as they are often found by chance in our simulation. However, it is known that some of such frequently occurring domain pairs do interact. Therefore, rather than immediately rejecting a pair with high p-value we consider additional evidence in terms of the so-called witness. A witness to a domain-domain interaction is a pair of interacting single domain proteins where one protein contains the first domain in the pair and the second protein contains the other. Given the reliability of each protein interaction in the network, one can estimate the conditional probability that the domain interaction is correct subject to observing a given set of witnesses. (For exact definitions and implementation details, see Methods).

Additional file 3 contains a table with the 1,399 domain pairs predicted by GPE to interact; they were chosen as those domain pairs that obtained an LP-score at least 0.60 and a pw-score less or equal to 0.01. A larger set of generalized domain scores is given in Additional file 4, which contains 7,554 generalized domain pairs that had LP-scores at least 0.50, regardless of their pw-scores. We point out, that if a domain pair occurs only once and this occurrence is in the context of an interaction between two single-domain proteins, the expected score of such a domain pair is 0.5. The results of predictions as functions of the network reliability, pw-threshold and LP-threshold are provided in Additional files 5 and 6. Following the IB approach, we excluded Pfam-B domains from this part of the study.

### The role of co-occurring domains in mediating protein interactions

To evaluate the role of co-occurring domains in mediating protein interactions represented by the network, we first computed the percentage of co-occurring domains in the iPfam benchmark set and in the sets of predicted domain interactions. Table 1 summarizes the results for PE (Original) and GPE (Generalized) predictions and the iPfam Benchmark Set (B). The iPfam benchmark set contains 61.8% of co-occurring (generalized) domains pairs (62.1% before introducing supra-domains). The percentage of co-occurring domains in predicted interactions was significantly lower than in the iPfam Benchmark Set (15.3% for the original and 22.9% for the generalized set) but still higher than expected by chance (4.6% and 4.4% respectively). Next, we tested if the difference in the level of enrichment in co-occurring domains in the iPfam benchmark set and in the predicted sets could be a result of artifacts in the prediction method. To do this we computed, under the assumption that protein interactions are mediated by domain interactions, the upper bound for the percentage of co-occurring domains in any set of interacting domain pair explaining the network. This computation is performed as follows. Assume that all co-occurring domains that could interact do interact. To estimate the upper bound on the fraction of co-occurring domains among interacting domains we need to compute a lower bound on the number of domain interactions needed to explain all interactions in the network. Note, that if the solution to our linear program was restricted to be integer, the optimum value of the objective function (the function optimized by the linear program) computed under the assumption that all domains are treated equally (thus without a bias towards co-occurrence) would give exactly the smallest possible number of domain pairs needed to explain the network. Since we don't restrict ourselves to an integer solution, the optimal value of the objective function could be smaller and therefore only provides a lower bound for the minimal number of interacting domain pairs. The resulting values were approximately 11,100 and 11,400 for the generalized and the original version, respectively. Thus, given the number of co-occurring domains is 1,146 (respectively 1,343 for the original domain definition) this implies that at most 10.3% (respectively 11.8%) of the interacting domain pairs could be co-occurring. This percentage depends on the assumed reliability of the network (here 0.5) and decreases with the increase of that reliability.

**Table 1:** Analysis of co-occurring domain pairs in the dataset and predictions

**ANALYSIS OF CO-OCCURRING PAIRS**				
		**All Pairs**	**Co-occur**	**Percentage**
**Potential Contacts**	**Original**	29,364	1,343	4.6%
	**Generalized**	26,113	1,146	4.4%
**Benchmark Set (B)**	**Original**	783	486	62.1%
	**Generalized**	691	427	61.8%
**Pairs Predicted**	**Original**	1,852	284	15.3%
	**Generalized**	1,399	321	22.9%
**Predicted ∩ B**	**Original**	230	182	79.1%
	**Generalized**	239	173	72.4%

The survey of the predicted domain interactions revealed that the percentage of co-occurring domains among predicted domain pairs was 15.3% for the original PE method and 22.9% for the generalized method (the statistics for different network reliability values and LP thresholds are given in the Additional file 6). The cost of a co-occurring domain pair has been initially set to 0.95 and the cost of all other domain pairs to 1.0. Decreasing further the cost of co-occurring domains did not influence the results significantly, even in the case where the co-occurring domains were assigned negligible costs. This suggests that the increase in the number of co-occurring domain pairs is obtained mainly by breaking ties in their favor. This survey of the predicted domain interactions provides an alternative estimate on the percentage of co-occurring domains in which, by selecting only highly scoring domains, we bypassed the requirement that all protein interactions had to be explained. However, this second estimate assumes that our predictions approximate the reality.

Finally, we tested the possibility that all the enrichment of co-occurring domains in the predicted interacting domains is exclusively due to iPfam domain pairs present in the prediction. By repeating the calculations with iPfam excluded, we found that, for the PE method, the percentage of co-occurring domains among the remaining predicted domain interactions is twice as big as expected by chance. This number was six times as big as the background for the predictions obtained by the GPE method that assigns smaller cost to co-occurring domains.

### Recovery of homodimers in the predictions

In a recent work, Itzhaki and colleagues observed that interacting homologous domains are overrepresented in crystal structures of interacting domains [37]. Therefore, we sought to investigate the presence of homodimers among our predictions. (Table 2 presents the detailed numbers.) We found that the ratio of homodimers in the set of all potentially interacting domain pairs is relatively small (2.5%). However, among the 1,399 domain pairs predicted by GPE, 203 pairs (14.5%) belong to that class. Hence, consistent with the findings of Itzhaki and colleagues, homodimers are significantly overrepresented in the set of predicted domain interactions. The enrichment remains significant after excluding iPfam domains: 5% of all predicted interactions that are not in iPfam are homodimers.

**Table 2:** Analysis of homodimers in the dataset, in GPEbenchmark set, and also among pairs predicted by GPE.

**ANALYSIS OF HOMODIMERS**			
	**All Pairs**	**Homodimers**	**Percentage**
**Potential Contacts**	26,113	656	2.5%
**Pairs Predicted**	1,399	203	14.5%
**Benchmark Set (B)**	691	383	55.4%
**Predicted ∩ B**	239	145	60.7%

Next, using the same method as in the case of the co-occurring domains, we found that under the assumption that protein interactions are mediated by domain interactions, at most 656/11100 = 5.9% of these interactions could potentially be homodimers. This fraction is much smaller than observed in the iPFAM benchmark set (55.4%).

### Analysis of top-ranked predictions

The seventy top-scoring pairs predicted with GPE are listed in Table 3. Most of the pairs in that list have multiple witnesses in the protein interaction network, but seven of them do not have any witnesses (thus always occur in the context of other domain pairs providing putative explanation, and therefore are called *difficult *in [2]), and all but one of those seven are confirmed by iPfam crystal structures.

**Table 3:** The seventy top-scoring domain pairs with pw-score at most 0.01.

**ORIG domA**	**ORIG domB**	**LP_score**	**pw_score**	**iPFAM**	**Witns**
7tm_1	IL8	1	0	0	80
AAA	AAA	1	0	1	36
AAA	PCI	1	0	0	18
Ank	RHD	1	0	1	0
Cpn60_TCP1	WD40	1	0	0	50
Cyclin_N	Pkinase	1	0	1	24
LSM	LSM	1	0	1	76
Pkinase	zf-C2H2	1	0	0	22
Pkinase	Pkinase	1	0	1	60
Pkinase	WD40	1	0	0	30
Proteasome	Proteasome	1	0	1	36
RNase_PH	RNase_PH	1	0	1	0
RNase_PH	RNase_PH_C	1	0	1	0
RRM_1	RRM_1	1	0	1	70
RRM_1	WD40	1	0	0	21
RRM_1	zf-C2H2	1	0	0	23
WD40	WD40	1	0	1	38
zf-C2H2	zf-C2H2	1	0	1	106
UQ_con	zf-C3HC4	1	0.00001	1	17
Pkinase	RRM_1	1	0.00002	0	16
TNF	TNFR_c6	1	0.00002	1	16
GTP_CDC	GTP_CDC	1	0.00003	0	15
Homeobox	Pkinase	1	0.00003	0	15
GDI	Ras	1	0.00006	1	14
PCI	PCI	1	0.00006	0	14
TPR	zf-C2H2	1	0.00006	0	14
LSM	WD40	1	0.00012	0	13
Metallophos	Proteasome	1	0.00012	0	13
AAA	WD40	1	0.00024	0	12
HLH	HLH	1	0.00024	1	12
Ras	Yip1	1	0.00024	0	12
WD40	zf-C2H2	1	0.00024	0	12
Hrf1	Ras	1	0.00049	0	11
Metallophos	efhand	1	0.00049	1	11
TPR	WD40	1	0.00049	0	11
Brix	WD40	1	0.00098	0	10
Homeobox	Homeobox	1	0.00098	1	10
Homeobox	zf-C2H2	1	0.00098	0	10
HSP70	Pkinase	1	0.00098	0	10
KH_1	WD40	1	0.00098	0	10
LSM	RRM_1	1	0.00098	0	10
LSM	Nop	1	0.00098	0	10
Metallophos	Pkinase	1	0.00098	0	10
PCI	Proteasome	1	0.00098	0	10
AAA	TPR	1	0.00195	0	9
Ank	Pkinase	1	0.00195	1	9
Chitin_bind_4	Chitin_bind_4	1	0.00195	0	9
E2F_TDP	SUPRDOM8	1	0.00195	0	9
HSP70	WD40	1	0.00195	0	9
KH_1	RRM_1	1	0.00195	0	9
LSM	zf-C2H2	1	0.00195	0	9
PH	Pkinase	1	0.00195	0	9
Pkinase	Rad50_zn_hook	1	0.00195	0	9
Pkinase	efhand	1	0.00195	0	9
Pkinase	TPR	1	0.00195	0	9
Histone	WD40	1	0.00391	0	8
Pkinase	Ras	1	0.00391	0	8
zf-C3HC4	zf-C3HC4	1	0.00391	0	8
HATPase_c	HATPase_c	1	0.005	1	1
SNARE	SNARE	1	0.005	1	1
efhand	efhand	1	0.00781	1	7
Homeobox	WD40	1	0.00781	0	7
PP2C	Pkinase	1	0.00781	0	7
RRM_1	TPR	1	0.00781	0	7
Zn_clus	Zn_clus	1	0.00781	1	7
HSP90	Pkinase	0.999	0	0	0
RHD	RHD	0.999	0	1	0
RHD	TIG	0.999	0	1	0
TIG	TIG	0.999	0	1	0
KE2	Prefoldin	0.999	0.00098	1	10

**Figure 1:**
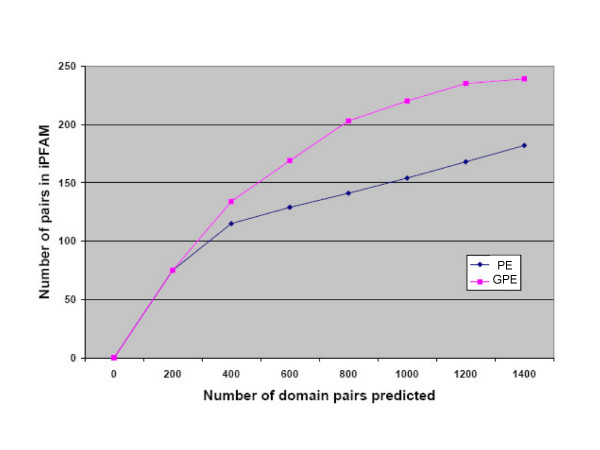
Benchmark pairs among top-scoring predictions.

**Figure 2:**
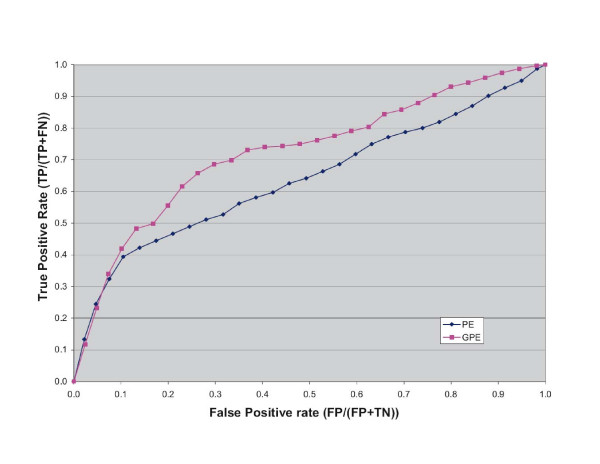
ROC Curves of GPE and PE.

First, we analyzed the results of GPE by assessing the retrieval of benchmark iPfam domain pairs among the top-ranked pairs in the list. Counting the predicted iPfam domains provides an assessment of quality of domain interaction prediction that is far from ideal, since only a small percentage of interacting domains are captured by crystallization experiments. Furthermore, according to our estimations, what is captured is a biased sample of interactions in the network. Nevertheless, we expect the top-scoring interactions to be enriched in iPFAM domain pairs. An assessment of the retrieval of benchmark pairs among the top-scoring pairs predicted by GPE and PE is given in Figure 1. The increased recovery rate of iPfam domains by the GPE method is measurable, although not overwhelming. The performances of the two methods measured with ROC curves are shown in Figure 2.

### Comparison with other methods

To compare GPE to the top methods, we use each method to predict the mediating domain pair(s) of a given protein interaction. The domain pair(s) with the highest score among the potentially interacting domain pairs is returned as the result of such prediction. Each protein interaction considered in this experiment contains one or more potential contacts in the iPfam benchmark set, which are assumed to be the true mediating pairs (such an experiment has been used before in a number of previous studies [2,25,38]). To make the comparison as fair as possible, the dataset used in this experiment is constrained by additional conditions detailed in the Methods Section, resulting in a set of 192 protein interactions, which are listed in Additional file 7.

**Figure 3:**
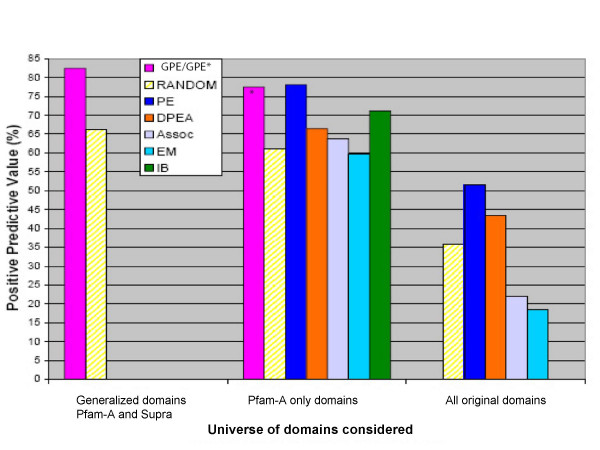
**Comparison of the Positive Predictive Values for several methods relative to the corresponding random performance.** The methods are grouped according to the domain definition. Note that performance of Random varies between the groups. GPE* denotes results obtained by projecting supra-domain from the GPE method is back into Pfam domains where the "children" domains inherit the scores from the supra-domain. A more formal comparison method from different groups and relies on counting how often each of them over/under-performed the corresponding random selection and is described in the text. The performance of GPE and PE was identical while their desistance to the next closed method was statistically significant.

The results of the accuracy represented by the positive predictive value (PPV= TP/(TP+FP)) of the above experiment are shown in Figure 3. Note that the performance of Random (choosing a mediating pair by chance) varies with the considered definition of a domain. GPE performs about 16 percentage points above

To evaluate the statistical differences between the methods, we computed for each method the number of times it wins and loses relative to Random and compared the corresponding fractions. By this measure the performance of PE and GPE was non-distinguishable (note that although the difference between the PPV values of GPE and PE is measureable, introducing supra-domains makes it easier for Random to guess the solution). The difference between the parsimony based methods and the next best method, the Integrative Bayesian method (IB) was, however, statistically significant (p-value < 0.005). This is interesting, since the IB scores were defined based on a wider range of information [3]. Due to the similar performance of GPE and IB, we were interested in determining which type of pairs, if any, were recovered by SPE and not by IB. Analysis of domain pairs predicted by our method and missed by IB suggests three reasons for unique selections made by our approach: using witnesses count, concatenating indistinguishable domains, and the linear programming optimization itself. The first two could be incorporated in any prediction method. Under the assumption of no systematic bias towards false positives, multiple occurrences of single domain protein interactions in the given network should be considered as experimental evidence. Combining domains into supra-domains had both obvious and more subtle effects. After combining multi-domain chains of RNA polymerase (RNA_pol_Rbp2), the interaction of this supra-domain with RNA_pol_L was easily detected. Obviously, which of the domain pairs are actually involved in the interaction cannot be determined based on the network alone. Another example involves the Retinoblastoma-like protein consisting of domain pair RB_A and RB_B, neither of which is ever observed without the other in our data. After combining them into one supra-domain, we recovered a known interaction with the E2F transcription factor [39-41] Our method uncovered also another known interaction of the RB supra-domain, namely with Histone deacetylase domain [42]. Based on the scores assigned by IB to individual domain pairs, we can speculate that this particular interaction would also be predicted by that method, should the domains be merged. The third class of predictions that obtained high scores by our method but were missed by IB contains groups of specific interactions where one or both of the partners are frequently occurring domains (e.g., signaling domains, DNA binding domains, etc). This is the most difficult class to predict correctly since it contains domain pairs that interact only in a specific context. Here, again, the domain pair (Hormone Receptor, bZIP), predicted exclusively by GPE, is consistent with the literature [43]. These predictions should be attributed to the parsimony based formulation of the problem. Additional file 8 contains a list of the 50 top-scoring domain pairs predicted by GPE that were not predicted by IB.

### Impact of the presence of PFAM-B domains

Because interactions involving Pfam-B domains are not documented by crystal structures, any method that is benchmarked using crystal structure data achieves better accuracy when Pfam-B domains are excluded. To make all methods comparable, we excluded pairs involving Pfam-B domains from the analysis. However, for all methods for which we had the corresponding data, we examined how these methods are affected by including Pfam-B domains. The results are presented in Figure 3.

Since the difficulty of the problem decreases with exclusion of Pfam-B, one should assess the performance of a method in a given setting relative to a random selection under the same setting. We found that the performances of the Association and the EM methods are drastically reduced upon inclusion of Pfam-B (they become worse than Random), while the performances of the parsimony method and DPEA remain well above random. We note that both the DPEA method and the parsimonious explanation method attempt (in different ways) to find domain pairs that are most prominent in explaining the interaction of proteins in the network. It is also important to stress the fact that although EM and Association performed worse than Random on this test, it does not mean that they would not outperform Random on other measures. For example, for all these methods, it has been demonstrated that high scoring predictions are enriched in iPfam pairs.

## Conclusion

In this paper, we studied the utility of the parsimony approach in detecting interacting domain partners. Furthermore, we introduced several improvements to our earlier Parsimonious Explanation (PE) method [2]. In its generalized version (GPE) the method adjusts the granularity of the domain definition to the granularity of the input data set and permits domain interactions to have different costs.

We also studied the impact of including *versus *excluding Pfam-B domains from predictions. In general, there are no crystal structures documenting interactions between Pfam-B domains. Thus, any method benchmarked using crystal structures can only benefit from excluding Pfam-B domains from predictions. This is unfortunate; as the prediction of interactions involving those not so well-studied domains are also of great interest. Therefore, we considered the impact of including Pfam-B on parsimony, DPEA, EM, and the Association methods. We found that among those, only the parsimony and the DPEA retained performance better than random.

The new objective function employed in GPE allows for assigning different costs to different types of interactions. We used this feature of GPE to study the effect of assigning a lower cost to domain pairs involving co-occurring domains. Despite this low cost, only about 23% of predicted domain interactions were between co-occurring domains – much less than in the benchmark crystal structure data which included 62% of this type of interactions. To see if the difference between these propensities is not an artifact of our prediction method, we computed, under the assumption that protein interactions are mediated by domain interactions, a lower bound on the number of domain interactions needed to explain protein interactions in the network. This in turn allowed us to estimate that the fraction of co-occurring domains among all interacting domains is at most approximately 11%. This estimation is made under the assumption that protein interactions are mediated by domain interactions but it is independent of any prediction method.

We also investigated another interesting observation that was made about domain-domain interactions, based on crystal structure data: enrichment in homodomain interactions [37]. Keeping in mind that data collected based on crystal structures may not be representative of genome wide properties, we sought to take advantage of the high confidence predictions and test if the observation holds for this data. Indeed, we found a significant bias toward homodomain interactions (14.5%) but much smaller than what has been observed in crystal structure benchmark data (55.4%). Once again, using the same counting argument as for the co-occurring domains, we confirmed that these differences are a real phenomenon and not an artifact of the prediction method.

These findings parallel the previously established fact that PDB data is not representative of genome wide protein data [17,34,35]. We stress that our computations have been made under the assumption that protein interactions are mediated by domain interactions. While this assumption is made by most domain interaction prediction methods, one should keep in mind that this is a simplification. Domains may also interact with peptides that are not part of any known domain. Alternatively, it is also is quite possible that protein-protein interactions present in high throughput networks are not a representative sample of all protein interactions and have their own biases. Therefore our estimation should be treated as evidence of a difference in the frequencies of certain types of domain-domain interactions in the two sets: the iPFAM set and the set of domain interactions mediating protein interactions in our high throughput network and not necessarily as an absolute truth about domain interactions in nature.

## Methods

### Formulation of the parsimony method as an LP problem

Our implementation of the parsimony principle uses linear programming optimization (LP) to find the smallest weighted set of domain-domain interactions that explains all protein-protein interactions.

The implementation is similar to the original LP formulation for the PE method. Intuitively, the linear program formalizes the task of finding a smallest weighted set of domain pairs subject to the constraints that all protein interactions are "explained". Formally, there is a variable for each unique potentially interacting domain pair (that is, a domain pair (A, B) such that A ∈ P1, B ∈ P2, and proteins P1 and P2 interact in the network), which can take any real value between zero and one. Additionally, each domain pair has assigned a cost (a number between 0 and 1) so that the interaction types that are known to be biologically more likely obtain a lower cost. Each protein interaction in the network is represented by a constraint requiring that the sum of the values assigned to the potentially interacting domain pairs must be at least 1.0. The goal of the LP is to minimize the weighted sum of the values assigned to all variables. Formally, if *CO *is the set of domain pairs that co-occur in the architecture of some protein in the network, *NCO *is the set of pairs that do not co-occur, and PPI represents the set of the protein interactions in the network, we have:

$$Minimize\sum_{(i,j)\in NCO}x_{ij}+\alpha \sum_{(i,j)\in CO}x_{ij}$$

$$\begin{array}{cc} Subject\;to: & \forall (P_{1}P_{2})\in PPI(\sum_{(p,q)\in P_{1}P_{2}}x_{pq})\geq 1 \end{array}$$

Since, as discussed in section 2.2, the results were (statistically) indistinguishable for a wide range of values of *α*, as long as *α *< 1; we have arbitrarily set *α *= 0.95.

### LP-score and pw-score

As in the original PE formulation, GPE also takes into account the reliability of the edges in the protein interaction network. This is done by creating 1000 random variants of LP instances where each constraint is included with probability equal to the reliability of the corresponding interaction. The actual LP-score of a variable is taken as the average of the values over all runs. Throughout this work, that reliability is assumed to be 50% [12-15].

Additionally, we provide the pw-score, which combines the traditional p-value (probability of obtaining a score at least this high by chance) and the so called witness-score. A witness to a domain-domain interaction is a pair of interacting single domain proteins where one protein contains the first domain in the pair and the second protein contains the other. If a domain pair (*i, j*) has *w*(*i, j*) witnesses and the reliability of each witness is *r*, then the witness-score is (1 - *r*)^*w*(*i*,*j*)^. That is, the witness support is the probability that all the witnesses of a given pair are false, as a function of the network reliability. The p-value is estimated in an independent randomization experiment where 1000 networks are created with the same proteins (with the same domain compositions) and the same number of protein interactions, but the protein pairs in the networks are chosen at random. The two indicators are then combined together to generate the pw-score as follows.

*pw*_*score*(*i*, *j*) = min(*p*_*value*(*i*, *j*),(1 - *r*)^*w*(*i*,*j*)^)

### Data sets

We used the data set by Riley and colleagues [26] which has also been used by Guimarães and colleagues [2], and is available online with the earlier paper. The protein interaction pairs were originally taken from the DIP database [44], and the domain architecture of the proteins were produced by HMM profiles from Pfam.

Adjusting the granularity of the data set yielded 162 supra-domains, which all together replaced 368 of the original domains. Replacing each group of commonly occurring domains with the corresponding supra-domains yielded 2,529 domains, a reduction of 7.5% in the total number of original PFAM-A domains (2,735). The list of the 162 supra-domains with the original domains that they encompass is in Additional file 1.

To formulate the LP we used only potential domain interactions involving Pfam-A domains or supra-domains. That led to a drastic reduction in the number of variables in the LP to 26,113 (with Pfam-B domains included, that number was about 170,000). Accordingly, in the protein interaction network we considered only the 10,025 proteins that had at least one Pfam-A domain in their architecture, which also affected the size of the LP, since the number of constraints went down to 20,625 (the entire dataset contained 26,032 constraints).

The benchmark set was a subset of the domain pairs in the iPFAM database [36], version of December 2005. We included only interchain interactions. The granularity of the benchmark set was adjusted to the granularity of our domain definition following the principle that supra-domains inherit the interactions of the domains they contain. The 691 benchmark pairs are listed in Additional file 2.

To compare pairs of methods that use different domain definitions, we compared the performance of each method to the performance of the Random method for the same domain definition. For each method we computed the number of times it performs better or worse than the Random method. This in turn was quantified by computing the percentage of iPfam domains (if any) in the set of highest scoring domain pairs. The proportion of "wins" to "losses" was then compared using the (2-sided) Fisher test.

### The dataset for the comparison of all methods

To be able to include the IB method in the comparison of our method with others, we needed to restrict ourselves to the 25,352 domain pairs with likelihood greater than 0.0 listed by Lee and colleagues [3] (as the scores of other domain pairs have not been provided.) Although the total number of pairs in that list is comparable to our 26,113, the two sets contain only 5,500 domain pairs in common. An important difference is that the 25,352 domain pairs published by Lee and colleagues is a selected set of domain pairs (which they predict to be more likely to interact than other domains in their original set) while our set contains all domain pairs that could potentially form interacting domain pairs given our set of interacting proteins. Furthermore, while the IB set contains 2,080 iPfam pairs (8.2%), the GPE set has only 691 iPfam pairs (2.6%). The small size of the overlap and the different iPfam ratio in the data suggest that the two datasets are rather different, so, for the sake of fairness, we use a more rigorous setting, where we only consider protein pairs for which scores from all methods are available. Additionally, we require that each interacting protein pair has an iPfam pair as a possible explanation. Finally, we removed all redundancies from this set, that is, no two interacting protein pairs have the same domain architecture. That led to a set of 192 protein interactions; those interactions are listed in Additional file 7.

In the comparison of the methods, we used the scores reported by Riley and colleagues [26] for DPEA, EM and Association, and the scores published by Lee and colleagues [3] for the IB method.

## Authors' contributions

KG participated in the design of the study, developed the experiments, and drafted the manuscript. TP conceived the study, participated in the design of the study, and helped to draft the manuscript. Both authors read and approved the final manuscript.

## Supplementary Material

Additional file 1 **Mapping of Supra-domains to domains**. This file contains 162 rows, each containing one supra-domain name followed by the names of the original domains included in it, separated by a blank character. Click here for file

Additional file 2 **Set of gold standard pairs**. This file contains the 691 generalized domain pairs in the gold standard set. Each line contains the names of two domains that comprise a pair, separated by a TAB character. Click here for file

Additional file 3 **GPE predictions**. This file contains a heading line and 1399 rows with the GPE predicted domain pairs ordered by LP-score. All pairs listed have LP-score ≥ 0.60 and pw-score ≤ 0.01. Each row contains five fields: Generalized Dom A, Generalized Dom B, GPE LP-score, GPE pw-score, and Gold Std (1 if pair is in GPE benchmark set, 0 otherwise). Click here for file

Additional file 4 **GPE LP-scores ≥ 0.50**. This file contains the 7,554 generalized domain pairs that had GPE LP-score ≥ 0.50, regardless of their pw-scores. Each row contains five fields: Generalized Dom A, Generalized Dom B, GPE LP-score, GPE pw-score, and Gold Std (1 if pair is in GPE benchmark set, 0 otherwise). Click here for file

Additional file 5 **Impact of pw-score cut-off on the quality of predictions**. This file contains the numbers of domains pairs predicted and the fraction of those that are in the benchmark set, for different values of pw-score cut-off. As the cut-off becomes more stringent, the accuracy of the predictions improves. Click here for file

Additional file 6 **Impact of reliability cut-off on the quality of predictions**. This file contains five tables for five different reliability cut-offs, showing the number of predictions and the number of predicted pairs in the benchmark set as a function of LP-score cut-off. Click here for file

Additional file 7 **Set of protein-protein interactions used to compare methods**. This file contains the 192 interacting protein pairs used in the experiment of predicting the mediating domain pairs, used to compare GPE with other state-of-the-art methods. Each line contains two integer numbers, separated by a TAB character, that represent the DIP identifiers of the interacting proteins. Click here for file

Additional file 8 **Pairs Predicted By GPE and not by IB**. This file contains the 50 top-scored original domain pairs that were predicted by GPE but had IB-score < 4.0. Each row contains six fields: ORIGdom A, ORIGdom B, GPE LP-score, GPE pw-score, IB-score, and number of Witnesses of that pair in GPE dataset in Generalized format. Click here for file
